# A Retrospective Study on the Use of Chinese Patent Medicine in 24 Medical Institutions for COVID-19 in China

**DOI:** 10.3389/fphar.2020.574562

**Published:** 2020-11-30

**Authors:** Nan Zhang, Nannan Shi, Siyu Li, Guoxiu Liu, Yonglong Han, Li Liu, Xin Zhang, Xiangwen Kong, Bihua Zhang, Wenpeng Yuan, Yi Liu, Deqiang Deng, Minxia Zheng, Ying Zhang, Lihua Li, Xiaoping Wang, Jiankun Wu, Xiaolan Lin, Hua Nian, Xiaohong Wu, Hua Wang, Fang Liu, Hongli Wang, Hongshun Wang, Ying Liu, Li Liu, Weixin Zeng, Manqin Yang, Yanping Wang, Huaqiang Zhai, Yongyan Wang

**Affiliations:** ^1^ Beijing University of Traditional Chinese Medicine, Beijing, China; ^2^ China Academy of Chinese Medical Sciences, Beijing, China; ^3^ Shanghai Jiao Tong University Affiliated Sixth People’s Hospital, Shanghai, China; ^4^ Hospital of Chengdu University of Traditional Chinese Medicine, Sichuan, China; ^5^ Integrated Hospital of Traditional Chinese Medicine, Southern Medical University, Guangdong, China; ^6^ Beijing University of Chinese Medicine Third Affiliated Hospital, Beijing, China; ^7^ Beijing Hospital, Beijing, China; ^8^ Shenzhen People’s Hospital, Guangdong, China; ^9^ Traditional Chinese Medicine Hospital of Urumqi, Xinjiang, China; ^10^ Zhejiang Provincial Hospital of Traditional Chinese Medicine, Zhejiang, China; ^11^ Eye Hospital, China Academy of Traditional Chinese Medicine, Beijing, China; ^12^ The First Affiliated Hospital of Anhui University of Traditional Chinese Medicine, Anhui, China; ^13^ Shaanxi Provincial Hospital of Traditional Chinese Medicine, Shaanxi, China; ^14^ Beijing Hospital of Traditional Chinese Medicine, Capital Medical University, Beijing, China; ^15^ Xuanwu Hospital of Capital Medical University, Beijing, China; ^16^ Yueyang Hospital of Integrated Traditional Chinese and Western Medicine, Shanghai University of Traditional Chinese Medicine, Shanghai, China; ^17^ Affiliated Hospital of Shanxi University of Traditional Chinese Medicine, Shanxi, China; ^18^ The Second Affiliated Hospital of Changchun University of Chinese Medicine, Jilin, China; ^19^ First Teaching Hospital of Tianjin University of Traditional Chinese Medicine, Tianjin, China; ^20^ Gansu Provincial Hospital of Traditional Chinese Medicine, Gansu, China; ^21^ Affiliated Hospital of Jiangxi University of Traditional Chinese Medicine, Jiangxi, China; ^22^ Peking University Third Hospital, Beijing, China; ^23^ Shuguang Hospital Affiliated to Shanghai University of Traditional Chinese Medicine, Shanghai, China; ^24^ Beijing Shijitan Hospital, Capital Medical University, Beijing, China; ^25^ The Second Affiliated Hospital of Anhui University of Traditional Chinese Medicine, Anhui, China

**Keywords:** Chinese patent medicine, COVID-19, application regularity, correlative factor, retrospective analysis

## Abstract

**Objective:** This research aims to analyze the application regularity of Chinese patent medicine during the COVID-19 epidemic by collecting the names of the top three Chinese patent medicines used by 24 hospitals in 14 provinces of China in four time periods (January 20–22, February 16–18, March 01–03, April 01–03, 2020), and explore its contribution to combating the disease.

**Methods:** 1) We built a database of the top three Chinese patent medicines used by 24 hospitals. 2) The frequency and efficacy distribution of Chinese patent medicine were analyzed with risk areas, regions, and hospitals of different properties as three factors. 3) Finally, we analyzed the differences in the use of heat-clearing and non-heat-clearing medicines among the three factors (χ^2^ test) and the correlation between the Chinese patent medicine and COVID-19 epidemic (correlation analysis) with SPSS 23.0 statistical software.

**Results:** 1) The heat-clearing medicine was the main use category nationwide during January 20–22, 2020. Meanwhile, there was a significant difference in the utilization rate of heat-clearing and non-heat-clearing medicine in different risk areas (*p* < 0.01). 2) The variety of Chinese patent medicine was increased nationwide during February 16–18, 2020, mainly including tonics, blood-activating and resolving-stasis, and heat-clearing medicines. Meanwhile, there was a significant difference in the utilization rate of heat-clearing and non-heat-clearing medicine in the southern and northern regions (*p* < 0.05). 3) Tonics, and blood-activating and resolving-stasis medicines became the primary use categories nationwide during March 01–03, 2020. 4) The tonics class, and blood-activating and resolving-stasis medicine were still the primary categories nationwide during April 01–03, 2020. Meanwhile, there was a significant difference in the utilization rate of heat-clearing and non-heat-clearing medicine in different risk areas (*p* < 0.01).

**Conclusion:** Chinese patent medicine has a certain degree of participation in fighting against the COVID-19. The efficacy distribution is related to the risk area, region, and hospital of different properties, among which the risk area is the main influencing factor. It is hoped that future research can further collect the application amount of Chinese patent medicine used in hospitals all over the country, so as to perfectly reflect the relationship between Chinese patent medicine and the epidemic situation.

## Introduction

COVID-19 is a contagious respiratory disease caused by severe acute respiratory syndrome coronavirus 2 (SARS‐CoV‐2), which was named by the coronavirus study group of the International Committee on Taxonomy of Viruses on February 11, 2020 (Sun et al., 2020). Currently, this epidemic disease has spread all around the world. The number of cumulative confirmed cases and existing confirmed cases in the countries except for China still show a continuous growth trend (World Health Organization, 2020), so the global pandemic remains severe.

From the Western Han Dynasty to the late Qing Dynasty, there were at least 321 large plagues in Chinese history. Therefore, Chinese history also contains a history of traditional Chinese medicine (TCM) against plagues. Faced with the SARS in 2003, China set up two independent “TCM Zone”, which achieved favorable results with the combination of Chinese and Western treatment. In the face of the COVID-19 pandemic, there is still a lack of effective drugs in the world. COVID-19 meeting of the Central Committee of China’s Leading Group request: strengthen the integration of TCM and western medicine, promote the whole process of the deep intervention of TCM diagnosis and treatment, and extend the effective TCM prescription and Chinese patent medicine (Wang et al., 2020).

There are four main aspects of TCM’s participation in the fight against COVID-19 (Zhang, 2020). First, providing TCM decoction to four quarantined groups of people, such as suspected and confirmed cases. Second, establishing Fangcang hospital, where nearly 10,000 patients almost entirely use TCM, and the coverage rate reached 95%. Third, for severe and critical patients, TCM also played an auxiliary role in improving oxygenation level and suppressing inflammatory factor storms. Finally, promoting recovery and reducing sequelae. TCM can remove residual evil, support vital qi, promote the absorption of pulmonary inflammation, and improve immune function. The proportion of TCM participating in the treatment of Hubei related hospitals was more than 2/3. The clinical practice data showed that the treatment of COVID-19 with integrated TCM and western medicine is effective (Yuan et al., 2020). TCM has shown remarkable effects in relieving fever symptoms, controlling disease progression, preventing disease transmissibility, reducing hormone dosage, decreasing complications, and preventing drug resistance (Chen et al., 2020).

The most obvious changes in the “sixth Trial Version of the Guidelines for the Diagnosis and Treatment of COVID-19” and later versions issued by the National Health Commission of China are the increased proportion of TCM therapeutic regimen, and the recommendation of Chinese patent medicine in different courses of COVID-19, especially the usage of TCM injections used for severe and critical patients. Twelve Chinese patent medicines are recommended for use in different stages of COVID-19 in the “seventh Trial Version of the Guidelines for the Diagnosis and Treatment of COVID-19” (National Health Commission National Administration of Traditional Chinese Medicine, 2020). Some studies have shown that Chinese patent medicines can significantly reduce the clinical manifestations of COVID-19 and play their pharmacological role in various mechanisms (Wang et al., 2020b; Zhang et al., 2020b).

This research aims to investigate the use of Chinese patent medicines used by 24 third-grade class-A hospitals in 14 provinces or cities of China during the epidemic from January to April, and analyze the usage characteristics, so as to have an in-depth understanding of the Chinese patent medicines’ participation and the related factors affecting its usage during the COVID-19 epidemic.

## Materials and Methods

### Data Sources

**FIGURE 1:**
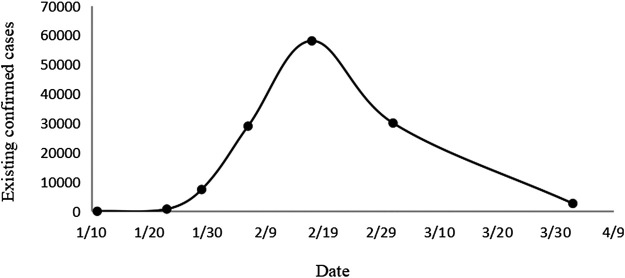
Trends in the number of confirmed cases over time.

Data of the name of Chinese patent medicine ranked top three used in 24 third-grade class-A hospitals in four time periods were collected. The four periods are January 20–22, February 16–18, March 01–03, April 01–03, 2020. The 24 hospitals are distributed in 14 provinces or cities of China (Beijing, Tianjin, Jilin Province, Shanxi Province, Shaanxi Province, Gansu Province, Xinjiang Province, Hubei Province, Zhejiang Province, Guangzhou Province, Anhui Province, Shanghai Province, Jiangxi Province, Sichuan Province). The above four time points are distributed in the initial stage, the highest peak, the fastest decline stage, and the end-stage, respectively, of the curve of the existing confirmed cases of the COVID-19 in China, aiming to fully reflect the drug use in all stages (Figure 1).

### Statistical Analysis

Data were analyzed with SPSS 23.0 statistical software. Chi-square (χ^**2**^) test was conducted when analyzing the difference of three factors in the frequency of heat-clearing and non-heat-clearing medicine. Regression analysis was taken when exploring the correlation between three factors and the epidemic situation. To avoid the influence of the uneven data on the analysis results, the frequency is weighted according to the proportion of the hospital with different properties when analyzing the hospital factor. Values of *p* < 0.05 and *p* < 0.01 were considered statistically significant differences and extremely significant differences separately.

### Exclusion Criteria

1. Specialist medicines;
2. The medicines with an obscure name.

Medicines with the same ingredient but different dosage forms are considered to be the same type.

### Classification Criteria of Three Factors

**FIGURE 2:**
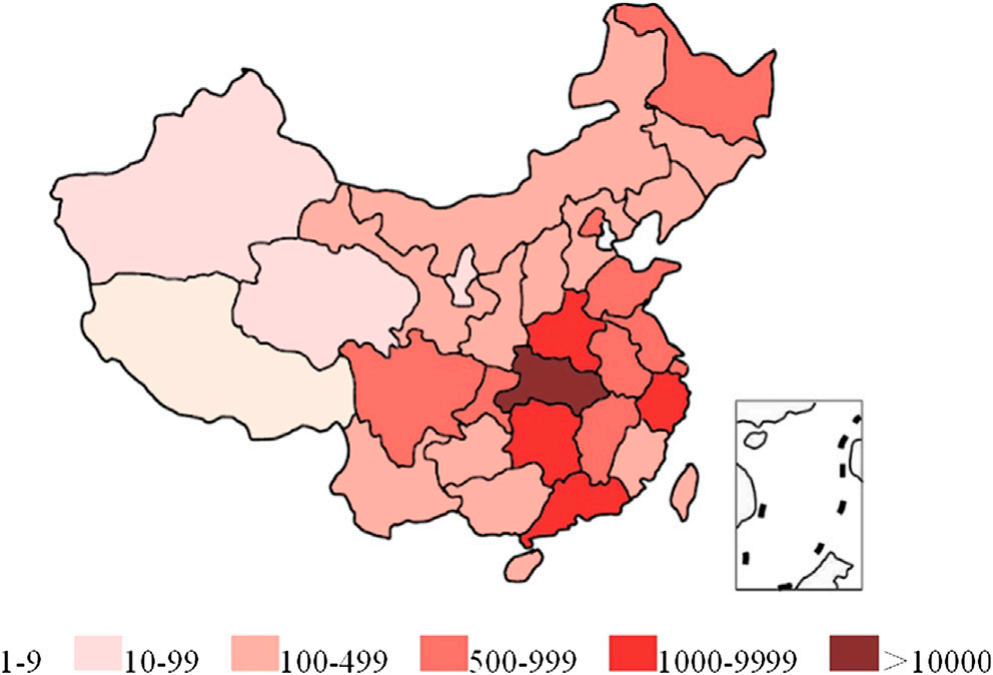
Six levels of confirmed cases in China.

Risk area classification criteria: Risk regions were divided into high-risk areas (cumulative confirmed cases >500) and low-risk areas (cumulative confirmed cases <500). According to the distribution of COVID-19 up to April 14, 2020, China was divided into six levels according to the accumulated confirmed cases (as shown in Figure 2). We select the median 500 as the boundary of the high and low-risk areas based on the severity of the epidemic at that time.

Region classification criteria: According to the south or north of the Qinling Mountain-Huaihe River Line, the areas within the statistical scope are divided into the southern region and the northern region. Qinling Mountain-Huaihe River line is currently recognized as China’s north-south geographical boundary. There were many differences on both sides of this line in the natural conditions, agricultural production, geographical features, and people’s living customs (Sheng, 2008).

Hospital classification criteria: According to the official website of the hospitals and the management system of the National Administration of TCM, 24 hospitals are divided into traditional Chinese medical hospitals (TCM hospitals) and Western medical hospitals (note: integrative medicine hospitals are included in TCM hospitals for their same TCM treatment department setting).

### Related Concepts

1. TCM includes Chinese medicinal decoction pieces/TCM decoction and Chinese patent medicine.
2. Chinese patent medicine is a kind of TCM product which is processed into a certain dosage form according to the prescription and preparation technology under the guidance of TCM theory and in order to prevent and treat diseases.
3. Heat-clearing medicine: The properties of heat-clearing medicine is cold, whcih can clear the body’s internal heat, including heat-clearing and detoxifying medicines, heat-clearing and fire-purging medicines, heat-clearing and damp-drying medicines, heat-clearing and blood-cooling medicines, et al.

Non-heat clearing medicine: The main function of non-heat clearing medicine is not to clear away heat, in this article refers to the tonifying deficiency medicines or activating blood and resolving stasis medicines.

## Results

### Data Statistics for January 20–22, 2020

#### Nationwide Data Statistics

The data of 24 hospitals nationwide were summarized and the name of Chinese patent medicines with a frequency of more than one was obtained. Among them, the top three Chinese medicines were Lianhua Qingwen granule (capsule), Lanqin oral liquid, and Jinhua Qinggan granule. It was shown that the medicine for clearing heat and removing toxicity is the main use category in the whole country from January 20 to January 22 (specific data is shown in Supplementary Table S1).

#### Data Statistics of Different Risk Areas

The usage frequency of Chinese patent medicine in high-risk and low-risk areas was counted (specific data is shown in Supplementary Tables S2, S3), respectively, and the distribution regularities of efficacy were analyzed (Tables 1, 2). By comparing the high-risk and low-risk areas, it can be seen that the high-risk areas are concentrated in heat-clearing medicines with a frequency as high as 86%, while the heat-clearing drugs accounting for only 37%. The analysis results showed that the usage frequency of heat-clearing medicines and non-heat-clearing medicines was significantly different between high-risk and low-risk areas (*p* < 0.01, Table 7). Therefore, the higher the risk level of the epidemic situation, the stronger the pertinence of drug types to the disease.

**TABLE 1:** Effect distribution of Chinese patent medicines in high-risk areas.

Name	Effect	Frequency	Percentage
Lianhua Qingwen granule (capsule)	CLLLLLlearing heat	20	87%
Lanqin oral liquid			
Jinhua Qinggan granule			
Huachansu capsule			
Antivirus oral liquid			
Banlangen granule			
Qingfei pill			
Feilike mixture			
Qingqiao Kangdu granule			
Jingyin mixture			
Chonglian oral liquid			
Compound shuanghua tablet			
Pudilan antiphlogistic oral liquid			
Huangkui capsule			
Compound Daqing granule			
Shufeng Jiedu capsule			
Xuanfei Zhisou mixture			
Compound Xianzhuli liquid			
Shiwei Longdanhua granule			
Maxing Huatan mixture			
Bailing capsule (tablet)	Tonifying deficiency	3	13%
Yupingfeng granule			
Shengxuebao mixture			

**TABLE 2:** Effect distribution of Chinese patent medicines in low-risk areas.

Effect	Frequency	Percentage (%)
Clearing heat	7	37
Tonifying deficiency	5	26
Activating blood and resolving stasis	7	37

#### Data Statistics of Different Regions

The usage frequency of Chinese patent medicine in southern and northern regions was counted (specific data is shown in Supplementary Tables S4, S5), respectively, and the distribution regularities of efficacy were analyzed (Table 3, 4). By comparing the northern region and southern region, it can be seen that the efficacy of Chinese patent medicines used in the southern region is relatively concentrated, with heat-clearing drugs (70%) as the main type, while the efficacy of Chinese patent medicines used in the northern region is relatively dispersed, with heat-clearing drugs (59%) as the first one, followed by the medicine for activating blood and resolving stasis and the medicine for tonifying deficiency. The analysis results showed that there was no significant difference in the frequency of heat-clearing drugs and non-heat-clearing drugs between southern and northern regions (*p* > 0.05, Table 7).

**TABLE 3:** Effect distribution of Chinese patent medicines in southern regions.

Name	Effect	Frequency	Percentage
Lianhua Qingwen granule (capsule)	Clearing heat	13	70%
Lanqin oral liquid			
Huachansucapsule			
Shufeng Jiedu capsule			
Antivirus oral liquid			
Pudilan antiphlogistic oral liquid			
Feilike mixture			
Maxing Huatan mixture			
Qingqiao Kangdu granule			
Jingyin mixture			
Chonglian oral liquid			
Xuanfei Zhisou mixture			
Compound Daqing granule			
Bailingcapsule (tablet)	Tonifying deficiency	5	30%
Shengxuebao mixture			
Yupingfeng granule			
Huaier granule			
Naoxintong capsule			

**TABLE 4:** Effect distribution of Chinese patent medicines in northern regions.

Name	Effect	Frequency	Percentage
Jinhua Qinggan granule	Clearing heat	17	59%
Lianhua Qingwen granule			
Lanqin oral liquid			
Antivirus oral liquid			
Banlangen granule			
Compound Xianzhuli liquid			
Shiwei Longdanhua granule			
Zukamu granule			
Qingfei pill			
Compound shuanghua tablet			
Honghua Qinggan thirteen pill			
Suhuang Zhike capsule			
Huangkui capsule			
Qingfei Huatan mixture			
Relinqing granule			
Modified Shuanghuanglian oral liquid			
Huachansu tablet			
Bailing capsule	Tonifying deficiency	6	21%
Shensong Yangxin capsule			
Jinshuibao pill			
Peiyuan Tongnao capsule			
Zhizhu Kuanzhong capsule			
Rougan Hepi pill			
Yinxing Mihuan oral liquid	Activating blood and resolving stasis	6	20%
Xuefu Zhuyu capsule			
Naoxintong capsule			
Compound Danshen dropping pill			
Guanxin Danshen dropping pill			
Danqi soft capsule			

The reason for the low utilization rate of heat-clearing medicines in northern regions may be related to the epidemic situation. The COVID-19 epidemic in southern China is extensive, so the used medicines focus on the prevention and treatment of pneumonia, while the used medicines in northern China focus on body regulation. Besides, considering that there is no significant difference in the northern and southern regions, so it is considered that the main factor affecting the drug use in January is epidemic risk grade without obvious direct correlation with geographical location.

#### Data Statistics of Hospitals of Different Properties

The usage frequency of Chinese patent medicine in TCM and western medical hospitals was counted (specific data is shown in Supplementary Tables S6, S7), respectively, and the distribution regularities of efficacy were analyzed (Table 5, 6). The comparison between TCM hospital and Western medical hospital shows that TCM hospitals mainly use heat-clearing medicines (60%), supplemented with Chinese patent medicines with different treatment principles such as tonifying deficiency, activating blood and resolving stasis. The purpose of treatment in western medical hospitals are relatively clear, and the frequency of using heat-clearing medicines is up to 78%. The analysis results showed that there was no significant difference between heat-clearing medicines and non-heat-clearing medicines between TCM hospitals and western medical hospitals (*p* > 0.05, Table 7).

**TABLE 5:** Effect distribution of Chinese patent medicines in traditional Chinese medicine hospitals.

Name	Effect	Frequency	Percentage
Lianhua Qingwen granule (capsule)	Clearing heat	22	60%
Jinhua Qinggan granule			
Shufeng Jiedu capsule			
Lanqin oral liquid			
Antivirus oral liquid			
Banlangen granule			
Compound Daqing granule			
Jingyin mixture			
Qingqiao Kangdu granule			
Pudilan antipyrotic oral liquid			
Chonglian oral liquid			
Huachansu capsule			
Qingfei pill			
Zukamu granule			
Huangkui capsule			
Qingfei Huatan mixture			
Maxing Huatan mixture			
Relinqing granule			
Honghua Qinggan thirteen pill			
Modified Shuanghuanglian oral liquid			
Xuanfei Zhisou mixture			
Suhuang Zhike capsule			
Bailingcapsule (tablet)	Tonifying deficiency	7	20%
Yupingfeng granule			
Shensong Yangxin capsule			
Shengxuebao mixture			
Jinshuibao pill			
Rougan Hepi pill			
Zhizhu Kuanzhong capsule			
Naoxintong capsule	Activating blood and resolving stasis	6	20%
Xuefu Zhuyu capsule			
Compound Danshen dropping pill			
Guanxin Danshen dropping pill			
Danqi soft capsule			
Yinxing Mihuan oral liquid			

**TABLE 6:** Effect distribution of Chinese patent medicines in western medical hospitals.

Name	Effect	Frequency	Percentage
Jinhua Qinggan granule	Clearing heat	7	78%
Lianhua Qingwen granule			
Compound Xianzhuli liquid			
Compound shuanghua tablet			
Huachansu capsule			
Shiwei Longdanhua granule			
Feilike mixture			
Huaier granule	Tonifying deficiency	2	22%
Peiyuan Tongnao capsule			

**TABLE 7:** Analysis of medication difference of three factors in January.

	Heat-clearing medicine (frequency)	Non-heat-clearing medicine (frequency)	*p*-value
High-risk area	20	4	0.002^a^
Low-risk area	7	12	
Sourthern region	13	5	0.345
Northern region	17	12	
Traditional Chinese medicine hospital	7	4	0.642
Western medical hospital	7	2	

a *p* < 0.01, High-risk area vs. Low-risk area.

The reasons for the differences in drug use may be related to the characteristics of different medical systems. TCM takes syndrome differentiation for treatment and body regulation as its primary treatment principles, so there were various kinds of medicines used. Western medical hospital emphasizes the symptomatic treatment, so the efficacy distribution of medicines was relatively narrow.

### Data Statistics for February 16–18, 2020

#### National Data Statistics

The data of 24 hospitals nationwide were summarized and the name of Chinese patent medicines with a frequency of more than one was obtained. Among them, the top three Chinese medicines are the Bailing capsule (tablet), compound Danshen dropping pill, and Lianhua Qingwen capsule (granule). From February 16 to 18, it was shown that the types of Chinese patent medicine had increased nationwide, such as tonifying deficiency, activating blood and resolving stasis, and heat-clearing medicines. Meanwhile, the utilization rate of heat-clearing drugs was lower than that in January (specific data is shown in Supplementary Table S8).

#### Data Statistics of Different Risk Areas

The usage frequency of Chinese patent medicine in high-risk and low-risk areas was counted (specific data is shown in Supplementary Tables S9, S10), respectively, and the distribution regularities of efficacy were analyzed (Table 8, 9). Comparing the high-risk and low-risk areas, the heat-clearing medicines in the high-risk areas still accounted for a large proportion (36%), while the low-risk areas were dominated by medicine for activating blood and resolving stasis (47%), with a significantly low utilization rate of heat-clearing medicines (18%). The analysis results showed that there was no significant difference between heat-clearing drugs and non-heat-clearing drugs in different risk areas (*p* > 0.05, Table 14).

**TABLE 8:** Effect distribution of Chinese patent medicines in high-risk areas.

Name	Effect	Frequency	Percentage
Lianhua Qingwengranule (capsule)	Clearing heat	15	36%
Xuebijing injection			
Xiyanping injection			
Jinhua Qinggangranule			
Lanqin oral liquid			
Chonglian oral liquid			
Jingyin mixture			
Banlangen granule			
Huangkui capsule			
Compound Daqing granule			
Maxing Huatan mixture			
Feilike mixture			
Compound Huangqi jiedu mixture			
Honghua Qinggan pill			
Babaodan capsule			
Compound Danshen dropping pill	Activating blood and resolving stasis	17	40%
Naoxintong capsule			
Linaoxin tablet			
Tiandan Tongluo capsule			
Yindan Xinnaotong soft capsule			
Tongluo Yiqi pill			
Naoshuantong capsule			
Xiaoshuan changyong capsule			
Qili Qiangxin capsule			
Xiaoshuan Tongluo capsule			
Xueshuan Xinmaining tablet			
Sanqi Shutong capsule			
Xiongdan capsule			
Ginkgo drop pill			
Xueshuantong granule			
Compound Xueshuantong capsule			
Shexiang Baoxin pill			
Bailingcapsule	Tonifying deficiency	10	24%
Jinshuibao pill			
Xinyuan capsule			
Yupingfeng granule			
Peiyuan Tongnao capsule			
Shenyan Kangfu tablet			
Zhenyuan capsule			
Kangfuxin liquid			
Yixinshu capsule			
Shenqi Gankang capsule			

**TABLE 9:** Effect distribution of Chinese patent medicines in low-risk areas.

Name	Effect	Frequency	Percentage
Lianhua Qingwen granule	Clearing heat	3	18%
Qingfei Huatan mixture			
Huangkui capsule			
Bailingcapsule	Tonifying deficiency	6	35%
Shensong Yangxin			
Qishen Yiqi drop pill			
Wenxin granule			
Jinshuibao pill			
Shenkangfu capsule 2			
Naoxintong capsule	Activating blood and resolving stasis	8	47%
Compound Danshen dropping pill			
Ginkgo tablet			
Naoxintong capsule			
Tongxinluo capsule			
Xuefu Zhuyugranule			
Yuxuebi capsule			
Compound Xueshuantong capsule			

#### Data Statistics of Different Regions

The usage frequency of Chinese patent medicine in the southern and northern regions was counted (specific data is shown in Supplementary Tables S11, S12), respectively, and the distribution regularities of efficacy were analyzed (Table 10, 11). Comparing the northern and southern region, the heat-clearing medicines were still dominant (41%) in the southern region. In northern regions, the medicine for activating blood and resolving stasis is the main type, and heat-clearing medicine accounts for the lowest proportion (17%). The analysis results showed that there were significant differences between heat-clearing medicines and non-heat-clearing medicines in different regions (*p* < 0.05, Table 14).

**TABLE 10:** Effect distribution of Chinese patent medicines in southern regions.

Name	Effect	Frequency	Percentage
Lianhua Qingwengranule (capsule)	Clearing heat	12	41%
Xiyanping injection			
Xuebijing injection			
Lanqin oral liquid			
Chonglian oral liquid			
Jingyin mixture			
Compound Huangqi jiedu mixture			
Compound Daqing granule			
Maxing Huatan mixture			
Feilike mixture			
Honghua Qinggan pill			
Babaodan capsule			
Compound Danshen dropping pill	Activating blood and resolving stasis	10	34%
Naoxintong capsule			
Ginkgo drop pill			
Shexiang Baoxin pill			
Yindan Xinnaotong soft capsule			
Naoshuantong capsule			
Tiandan Tongluo capsule			
Tongluo Yiqi pill			
Xiaoshuan changyong capsule			
Xiongdan capsule			
Bailing capsule	Tonifying deficiency	7	24%
Jinshuibao pill			
Yixinshu capsule			
Yupingfeng granule			
Shenqi Duotang oral liquid			
Shenyan Kangfu tablet			
Shenqi Gankang capsule			

**TABLE 11:** Effect distribution of Chinese patent medicines in northern regions.

Name	Effect	Frequency	Percentage
Lianhua Qingwen granule	Clearing heat	5	17%
Huangkui capsule			
Jinhua Qinggangranule			
Qingfei Huatan mixture			
Banlangen granule			
Naoxintong capsule	Activating blood and resolving stasis	14	48%
Compound Danshen dropping pill			
Ginkgo tablet			
Linaoxin tablet			
Compound Xueshuantong capsule			
Xuefu Zhuyugranule			
Xueshuantong granule			
Tongxinluo capsule			
Qili Qiangxin capsule			
Sanqi Shutong capsule			
Yuxuebi capsule			
Tiandan Tongluo capsule			
Xiaoshuan Tongluo capsule			
Xueshuan Xinmaining tablet			
Bailingcapsule	Tonifying deficiency	10	34%
Jinshuibao pill			
Xinyuan capsule			
Shensong Yangxin			
Kangfuxin liquid			
Zhenyuan capsule			
Wenxin granule			
Qishen Yiqi drop pill			
Shenkangfu 2 capsule			
Peiyuan Tongnao capsule			

Analyzing the reasons for the significant difference of drug use in February between the northern and southern regions, it was concluded that, the characteristics of drug use in different regions are revealed with the development and research of the COVID-19. Therefore, the region became the main influence factor of drug use in February.

#### Data Statistics of Hospitals of Different Properties

The usage frequency of Chinese patent medicine in TCM and western medical hospitals was counted (specific data is shown in Supplementary Tables S13, S14), respectively, and the distribution regularities of efficacy were analyzed (Table 12, 13). The comparison between TCM hospitals and western medical hospitals showed that the use of Chinese patent medicine in February in two kinds of hospitals was similar. Although both of them take medicine for activating blood and removing stasis as the primary use category, heat-clearing medicines still account for a large proportion. Analysis results showed that there was no significant difference between heat-clearing medicines and non-heat-clearing medicines in different hospitals (*p* > 0.05, Table 14).

**TABLE 12:** Effect distribution of Chinese patent medicines in traditional Chinese medicine hospitals.

Name	Effect	Frequency	Percentage
Lianhua Qingwengranule (capsule)	Clearing heat	12	32%
Huangkui capsule			
Xiyanping injection			
Xuebijing injection			
Maxing Huatan mixture			
Jingyin mixture			
Chonglian oral liquid			
Qingfei Huatan mixture			
Compound Daqing granule			
Honghua Qinggan pill			
Compound Huangqi jiedu mixture			
Babaodan capsule			
Compound Danshen dropping pill	Activating blood and resolving stasis	15	41%
Naoxintong capsule			
Compound Xueshuantong capsule			
Ginkgo tablet			
Tongxinluo capsule			
Tongluo Yiqi pill			
Linaoxin tablet			
Tiandan Tongluo capsule			
Naoshuantong capsule			
Xueshuantong granule			
Yuxuebi capsule			
Xiongdan capsule			
Shexiang Baoxin pill			
Xuefu Zhuyu granule			
Qili Qiangxin capsule			
Bailingcapsule (tablet)	Tonifying deficiency	10	27%
Jinshuibao pill			
Shensong Yangxin			
Shenqi Duotang oral liquid			
Wenxin granule			
Yupingfeng granule			
Qishen Yiqi drop pill			
Peiyuan Tongnao capsule			
Shenyan Kangfu tablet			
Shenkangfu capsule 2			

**TABLE 13:** Effect distribution of Chinese patent medicines in western medical hospitals.

Name	Effect	Frequency	Percentage
Lianhua Qingwen granule	Clearing heat	5	31%
Jinhua Qinggan granule			
Lanqin oral liquid			
Banlangen granule			
Feilike mixture			
Sanqi Shutong capsule	Activating blood and resolving stasis	6	38%
Tiandan Tongluo capsule			
Xiaoshuan Tongluo capsule			
Xueshuan Xinmaining tablet			
Linaoxin tablet			
Yindan Xinnaotong soft capsule			
Xinyuan capsule	Tonifying deficiency	5	31%
Jinshuibao pill			
Kangfuxin liquid			
Zhenyuan capsule			
Yixinshu capsule			

**TABLE 14:** Analysis of medication difference of three factors in February.

	Heat-clearing medicine (frequency)	Non-heat-clearing medicine (frequency)	*p-*value
High-risk area	15	27	0.172
Low-risk area	3	14	
Sourthern region	12	17	0.043^a^
Northern region	5	24	
Traditional Chinese medicine hospital	4	8	0.907
Western medical hospital	5	11	

a *p* < 0.05, Southern region vs. Northern region.

### Data Statistics for March 01–03, 2020

#### National Data Statistics

The data of 24 hospitals nationwide were summarized and the name of Chinese patent medicines with a frequency of more than one was obtained. Among them, the top four Chinese patent medicines are the Bailing capsule (tablet), compound Danshen dropping pill, Naoxintong capsule, Lianhua Qingwen granules (capsule). During March 01–03, medicines for tonifying deficiency, and activating blood and resolving stasis became the main categories nationwide (specific data is shown in Supplementary Table S27).

#### Data Statistics of Different Risk Areas

The usage frequency of Chinese patent medicine in high-risk and low-risk areas was counted (specific data is shown in Supplementary Tables S16, S17), respectively, and the distribution regularities of efficacy were analyzed (Table 15, 16). Comparing with the high-risk area and low-risk area, the utilization rate of heat-clearing medicines (33%) in the high-risk areas was about twice as much as that in the low-risk area (12%). The results showed that there was no significant difference between the use frequency of heat-clearing medicines and non-heat-clearing medicines in the high-risk and low-risk areas (*p* > 0.05, Table 21).

**TABLE 15:** Effect distribution of Chinese patent medicines in high-risk areas.

Name	Effect	Frequency	Percentage
Lianhua Qingwen granule (capsule)	Clearing heat	14	33%
Xiyanping injection			
Xuebijing injection			
Jinhua Qinggan granule			
Lanqin oral liquid			
Huangkui capsule			
Compound Huangqi jiedu mixture			
Compound Daqing granule			
Antivirus oral liquid			
Huachansucapsule			
Maxing Huatan mixture			
Honghua Qinggan pill			
Feilike mixture			
Zhenbao pill			
Bailing capsule (tablet)	Tonifying deficiency	15	34%
Jinshuibao pill			
Shensong Yangxin			
Shengxuebao mixture			
Shenqi Duotang oral liquid			
Dengzhan Shengmaicapsule			
Xinyuan capsule			
Wenxin granule			
Linglingcapsule			
Shenqi Gankang capsule			
Yishen Huashigranule			
Congrong Yishen granule			
Compound congrong Yizhicapsule			
Shenyan Kangfu tablet			
Shenshuaining capsule			
Compound Danshen dropping pill	Activating blood and resolving stasis	14	33%
Naoxintong capsule			
Ginkgo tablet (drop pill)			
Xintong oral liquid			
Xuefu Zhuyu capsule			
Compound Xueshuantong capsule			
Xueshuantong granule			
Qili Qiangxin capsule			
Sanqi Shutong capsule			
Xueshuan Xinmaining tablet			
Xiaoshuan changyong capsule			
Linaoxin tablet			
Tiandan Tongluo capsule			
Xiongdan capsule			

**TABLE 16:** Effect distribution of Chinese patent medicines in low-risk areas.

Name	Effect	Frequency	Percentage
Yifei Jiedu granule	Clearing heat	2	12%
Yichuanping capsule			
Compound Danshen dropping pill	Activating blood and resolving stasis	7	41%
Naoxintong capsule			
Yuxuebi capsule			
Danqicapsule			
Tongxinluo capsule			
Xuefu Zhuyu capsule			
Compound Xueshuantong capsule			
Bailing capsule	Tonifying deficiency	8	47%
Shensong Yangxin capsule			
Jinshuibao pill			
Shenkangfu 2 capsule			
Xianling Gubaocapsule			
Fufang Xuanju capsule			
Rougan Hepi pill			
Zhizhu Kuanzhong capsule			

#### Data Statistics of Different Regions

The usage frequency of Chinese patent medicine in the southern and northern regions was counted (specific data is shown in Supplementary Tables S18, S19), respectively, and the distribution regularities of efficacy were analyzed (Table 17, 18). It can be seen that the proportion of heat-clearing medicines in southern regions is the highest (40%). In the northern regions, the heat-clearing medicines account for the smallest proportion (20%), while activating blood and removing stasis is the category with the highest utilization rate (47%). The analysis results showed that there was no significant difference in the frequency of using heat-clearing medicines and non-heat-clearing medicines between southern and northern regions (*p* > 0.05, Table 21).

**TABLE 17:** Effect distribution of Chinese patent medicines in southern regions.

Name	Effect	Frequency	Percentage
Lianhua Qingwengranule (capsule)	Clearing heat	12	40%
Xuebijing injection			
Xiyanping injection			
Lanqin oral liquid			
Antivirus oral liquid			
Compound Huangqi jiedu mixture			
Compound Daqing granule			
Maxing Huatan mixture			
Huachansucapsule			
Honghua Qinggan pill			
Huangkui capsule			
Feilike mixture			
Bailingcapsule (tablet)	Tonifying deficiency	10	33%
Jinshuibao pill			
Shengxuebao mixture			
Dengzhan Shengmai capsule			
Lingling capsule			
Shenqi Duotang oral liquid			
Wenxin granule			
Congrong Yishen granule			
Yishen Huashigranule			
Shenqi Gankang capsule			
Naoxintong capsule	Activating blood and resolving stasis	8	27%
Compound Danshen dropping pill			
Ginkgo drop pill			
Xiaoshuan changyong capsule			
Tiandan Tongluo capsule			
Shenshuainingcapsule			
Shenyan Kangfu tablet			
Xiongdan capsule			

**TABLE 18:** Effect distribution of Chinese patent medicines in northern regions.

Name	Effect	Frequency	Percentage
Lianhua Qingwen granule	Clearing heat	6	20%
Jinhua Qinggan granule			
Yifei Jiedu granule			
Yichuanping capsule			
Huangkui capsule			
Zhenbao pill			
Compound Danshen dropping pill	Activating blood and resolving stasis	14	47%
Naoxintong capsule			
Compound Xueshuantong capsule			
Ginkgo tablet			
Danqi capsule			
Sanqi Shutong capsule			
Xueshuantong granule			
Tongxinluo capsule			
Xueshuan Xinmaining tablet			
Qili Qiangxin capsule			
Linaoxin tablet			
Yuxuebi capsule			
Xintong oral liquid			
Xuefu Zhuyu capsule			
Bailing capsule	Tonifying deficiency	10	33%
Shensong Yangxin			
Jinshuibao pill			
Xinyuan capsule			
Xianling Gubao			
Shenkangfu capsule2			
Fufang Xuanju capsule			
Compound congrong Yizhi capsule			
Zhizhu Kuanzhong capsule			
Rougan Hepi pill			

#### Data Statistics of Hospitals of Different Properties

The usage frequency of Chinese patent medicine in TCM and western medical hospitals was counted (specific data is shown in of Supplementary Tables S20, S21), respectively, and the distribution regularities of efficacy were analyzed (Table 19, 20). It can be seen that both TCM hospitals and Western medical hospitals tend to use medicines for activating blood and removing stasis, and tonifying deficiency. The results showed that there was no significant difference in the usage frequency of heat-clearing medicines and non-heat-clearing medicines between different hospitals (*p* > 0.05, Table 21).

**TABLE 19:** Utilization rate of Chinese patent medicine in traditional Chinese medicine hospitals.

Name	Effect	Frequency	Percentage
Lianhua Qingwengranule (capsule)	Clearing heat	10	25%
Xiyanping injection			
Xuebijing injection			
Compound Daqing granule			
Compound Huangqi jiedu mixture			
Maxing Huatan mixture			
Yichuanping capsule			
Yifei Jiedu granule			
Honghua Qinggan pill			
Huachansu capsule			
Compound Danshen dropping pill	Activating blood and resolving stasis	14	35%
Naoxintong capsule			
Compound Xueshuantong capsule			
Ginkgo drop pill (tablet)			
Danqicapsule			
Xuefu Zhuyu capsule			
Yuxuebi capsule			
Tongxinluo capsule			
Xiaoshuan changyong capsule			
Sanqi Shutong capsule			
Tiandan Tongluo capsule			
Xueshuantong granule			
Qili Qiangxin capsule			
Xiongdan capsule			
Bailingcapsule (tablet)	Tonifying deficiency	16	40%
Jinshuibao pill			
Shensong Yangxin			
Shenqi Duotang oral liquid			
Lingling capsule			
Shengxuebao mixture			
Wenxin granule			
Shenqi Gankang capsule			
Zhizhu Kuanzhong capsule			
Rougan Hepi pill			
Shenshuaining capsule			
Shenyan Kangfu tablet			
Shenkangfu capsule 2			
Fufang Xuanju capsule			
Xianling Gubao			
Yishen Huashi granule			

**TABLE 20:** Effect distribution of Chinese patent medicines in western medical hospitals.

Name	Effect	Frequency	Percentage
Lianhua Qingwen granule	Clearing heat	6	33%
Jinhua Qinggan granule			
Lanqin oral liquid			
Antivirus oral liquid			
Zhenbao pill			
Feilike mixture			
Compound Danshen dropping pill	Activating blood and resolving stasis	5	28%
Naoxintong capsule			
Linaoxin tablet			
Xueshuan Xinmaining tablet			
Xintong oral liquid			
Bailing capsule	Tonifying deficiency	7	39%
Dengzhan Shengmai capsule			
Jinshuibao pill			
Shensong Yangxin			
Congrong Yishen granule			
Compound congrong Yizhicapsule			
Xinyuan capsule			

**TABLE 21:** Analysis of medication difference of three factors in March.

	Heat-clearing medicine (frequency)	Non-heat-clearing medicine (frequency)	*p-*value
High-risk area	14	29	0.101
Low-risk area	2	15	
Sourthern region	12	18	0.091
Northern region	6	24	
Traditional Chinese medicine hospital	3	10	0.585
Western medical hospital	6	12	

### Data Statistics for April 01–03, 2020

#### National Data Statistics

The data of 24 hospitals nationwide were summarized and the name of Chinese patent medicines with a frequency of more than one was obtained. Among them, the top four proprietary Chinese medicines are the Bailing capsule, compound Danshen dropping pill, Naoxintong capsule, Jinshuibao tablet, and Lianhua Qingwen granules (capsules) (specific data is shown in Table 40 Supplementary Table S22).

#### Data Statistics of Different Risk Areas

The usage frequency of Chinese patent medicine in high-risk and low-risk areas was counted (specific data is shown in Supplementary Tables S23, S24), respectively, and the distribution regularities of efficacy were analyzed (Table 22, 23). The high-risk areas in April were still dominated by heat-clearing medicines. In the low-risk areas, the medicines for activating blood and removing stasis, and tonifying deficiency have occupied the majority of the commonly used medicines, while the utilization rate of heat-clearing medicines has decreased significantly, accounting for only 5%. The analysis results showed that the usage frequency of heat-clearing medicines and non-heat-clearing medicines was significantly different between high-risk and low-risk areas (*p* < 0.01, Table 28).

**TABLE 22:** Effect distribution of Chinese patent medicines in high-risk areas.

Name	Effect	Frequency	Percentage
Bailing capsule (tablet)	Clearing heat	15	39%
Lianhua Qingwen granule (capsule)			
Jinhua Qinggan granule			
Lanqin oral liquid			
Huachansucapsule			
Huangkui capsule			
Chonglian oral liquid			
Compound Daqing granule			
Jingyin mixture			
Feilike mixture			
Niuhuang Qingxin pill			
Longqing tablet			
Zhenbao pill			
Honghua Qinggan thirteen pill			
Weimaining capsule			
Jinshuibao pill	Tonifying deficiency	11	28%
Shengxuebao mixture			
Shensong Yangxin			
Longlu capsule			
Mingmu Yanggan pill			
Jiuwei Zhenxin granule			
Lishukang capsule			
Qiwei Wenyang capsule			
Congrong Yishen granule			
Wenxin granule			
Shenyan Kangfu tablet			
Naoxintong capsule	Activating blood and resolving stasis	13	33%
Compound Danshen dropping pill			
Maizhiling tablet			
Tiandan Tongluo capsule			
Huoxue Tongmaicapsule			
Salvia miltiorrhiza polyphenolic acid for injection			
Compound Xueshuantong capsule			
Xueshuan Xinmaining tablet			
Yindan Xinnaotong soft capsule			
Xiaoshuan Tongluo capsule			
Yuxuebi capsule			
Xiaoshuan changyong capsule			
Honghua Xiaoyao tablet			

**TABLE 23:** Effect distribution of Chinese patent medicines in low-risk areas.

Name	Effect	Frequency	Percentage
Lianhua Qingwen granule	Clearing heat	1	5%
Zhizhu Kuanzhong capsule	Tonifying deficiency	9	45%
Bailing capsule			
Shensong Yangxin			
Jinshuibao pill			
Fufang Xuanju capsule			
Gujin pill			
Kangfuxin liquid			
Xianling Gubao capsule			
Longlu pill			
Compound Danshen dropping pill	Activating blood and resolving stasis	10	50%
Naoxintong capsule			
Tongxinluo capsule			
Guanxin Danshen dropping pill			
Yinxing Mihuan oral liquid			
Xuefu Zhuyu capsule			
Yuxuebi capsule			
Danqi soft capsule			
Moxa stick			
Suxiao Jiuxin pill			

#### Statistics of Different Regions

The usage frequency of Chinese patent medicine in the southern and northern regions was counted (specific data is shown in Supplementary Tables S25, S26), respectively, and the distribution regularities of efficacy were analyzed (Table 24, 25). It can be seen that Chinese patent medicines mainly used in southern China are still heat-clearing medicine, followed by the medicine for tonifying deficiency, and activating blood and removing stasis. In northern China, the main category is the medicine for activating blood and removing stasis, followed by heat-clearing medicine and medicine for tonifying deficiency. The analysis results showed that there was no significant difference in the frequency of heat-clearing medicines and non-heat-clearing medicines between the southern and northern regions (*p* > 0.05, Table 28).

**TABLE 24:** Effect distribution of Chinese patent medicines in southern regions.

Name	Effect	Frequency	Percentage
Lianhua Qingwen capsule	Clearing heat	10	38%
Lanqin oral liquid			
Compound Daqing granule			
Chonglian oral liquid			
Jingyin mixture			
Feilike mixture			
Huachansu capsule			
Huangkui capsule			
Longqing tablet			
Weimaining capsule			
Bailingcapsule (tablet)	Tonifying deficiency	9	35%
Jinshuibao pill			
Shenyan Kangfu tablet			
Longlu capsule			
Lishukang capsule			
Qiwei Wenyang capsule			
Congrong Yishen granule			
Shengxuebao mixture			
Wenxin granule			
Compound Danshen dropping pill	Activating blood and resolving stasis	7	27%
Naoxintong capsule			
Honghua Xiaoyao tablet			
Qufeng Zhitong pill			
Tiandan Tongluo capsule			
Xiaoshuan changyong capsule			
Yindan Xinnaotong soft capsule			

**TABLE 25:** Effect distribution of Chinese patent medicines in northern regions.

Name	Effect	Frequency	Percentage
Lianhua Qingwen capsule	Clearing heat	8	23%
Jinhua Qinggan granule			
Huangkui capsule			
Lanqin oral liquid			
Huachansu capsule			
Zhenbao pill			
Honghua Qinggan thirteen pill			
Niuhuang Qingxin pill			
Jinshuibao pill	Tonifying deficiency	11	31%
Bailing capsule			
Kangfuxin liquid			
Shensong Yangxin			
Xianling Gubao capsule			
Longlu pill			
Gujin pill			
Fufang Xuanju capsule			
Mingmu Yanggan pill			
Jiuwei Zhenxin granule			
Zhizhu Kuanzhong capsule			
Naoxintong capsule	Activating blood and resolving stasis	16	46%
Compound Danshen dropping pill			
Yuxuebi capsule			
Compound Xueshuantong capsule			
Salvia miltiorrhiza polyphenolic acid for injection			
Xuefu Zhuyu capsule			
Danqi soft capsule			
Xueshuan Xinmaining tablet			
Tiandan Tongluo capsule			
Xiaoshuan Tongluo capsule			
Huoxue Tongmaicapsule			
Tongxinluo capsule			
Suxiao Jiuxin pill			
Guanxin Danshen dropping pill			
Yinxing Mihuan oral liquid			
Moxa stick			

#### Data Statistics of Hospitals of Different Properties

The usage frequency of Chinese patent medicine in TCM and western medical hospitals was counted (specific data is shown in Supplementary Tables S27, S28), respectively, and the distribution regularities of efficacy were analyzed (Table 26, 27). It can be seen from the comparison between TCM hospital and western medical hospital that the main treatment direction of TCM hospital is to tonify deficiency and promote circulation and remove stasis, which is in line with the characteristics of TCM for the recovery period. Although the western hospital still takes clearing heat as the primary treatment direction, the utilization rate of the other two kinds of medicine has increased, which is in agreement with the different stages of the epidemic situation in general. The analysis results showed that there was no significant difference between heat-clearing and non-heat-clearing medicines between two different types of hospitals (*p* > 0.05, Table 28).

**TABLE 26:** Effect distribution of Chinese patent medicines in traditional Chinese medicine hospitals.

Name	Effect	Frequency	Percentage
Lianhua Qingwen capsule (granule)	Clearing heat	9	24%
Chonglian oral liquid			
Jingyin mixture			
Huangkui capsule			
Compound Daqing granule			
Huachansu capsule			
Honghua Qinggan thirteen pill			
Longqing tablet			
Weimaining capsule			
Bailing capsule (tablet)	Tonifying deficiency	14	38%
Jinshuibao pill			
Shensong Yangxin			
Longlucapsule (pill)			
Zhizhu Kuanzhong capsule			
Shengxuebao mixture			
Wenxin granule			
Shenyan Kangfu tablet			
Xianling Gubao capsule			
Lishukang capsule			
Fufang Xuanju capsule			
Mingmu Yanggan pill			
Gujin pill			
Kangfuxin liquid			
Naoxintong capsule	Activating blood and resolving stasis	14	38%
Compound Danshen dropping pill			
Yuxuebi capsule			
Danqi soft capsule			
Honghua Xiaoyao tablet			
Salvia miltiorrhiza polyphenolic acid for injection			
Guanxin Danshen dropping pill			
Tongxinluo capsule			
Tiandan Tongluo capsule			
Xuefu Zhuyu capsule			
Compound Xueshuantong capsule			
Suxiao Jiuxin pill			
Yinxing Mihuan oral liquid			
Moxa stick			

**TABLE 27:** Effect distribution of Chinese patent medicines in western medical hospitals.

Name	Effect	Frequency	Percentage
Lianhua Qingwen granule	Clearing heat	7	41%
Lanqin oral liquid			
Jinhua Qinggan granule			
Huachansu capsule			
Niuhuang Qingxin pill			
Zhenbao pill			
Feilike mixture			
Qiwei Wenyang capsule	Tonifying deficiency	4	24%
Congrong Yishen granule			
Kangfuxin liquid			
Jiuwei Zhenxin granule			
Compound Danshen dropping pill	Activating blood and resolving stasis	6	35%
Huoxue Tongmaicapsule			
Xueshuan Xinmaining tablet			
Xiaoshuan Tongluo capsule			
Tiandan Tongluo capsule			
Yindan Xinnaotong soft capsule			

**TABLE 28:** Analysis of medication difference of three factors in April.

	Heat-clearing medicine (frequency)	Non-heat-clearing medicine (frequency)	*p-*value
High-risk area	15	24	0.006^a^
Low-risk area	1	19	
Sourthern region	10	16	0.186
Northern region	8	27	
Traditional Chinese medicine hospital	3	9	0.367
Western medical hospital	7	10	

a *p* < 0.01, High-risk area vs. Low-risk area.

**TABLE 29:** Utilization rate of heat-clearing medicines of three factors.

Date	Existing confirmed cases (average)	Whole nation	High-risk area	Low-risk area	Southern region	Northern region	Traditional Chinese medicine hospital	Western medical hospital
20–22	434	65%	86%	37%	70%	59%	60%	78%
16–18	57,918	31%	36%	18%	41%	17%	32%	31%
1–3	30,030	30%	33%	12%	40%	20%	25%	33%
1–3	1717	29%	39%	5%	38%	23%	24%	41%

*p* > 0.05, Existing confirmed cases vs. three factors.

## Discussion

According to the data and analysis results of this study, it is considered that the analysis method (χ^**2**^ test) matches the type of data and research purpose (significant difference), and more scientific and reasonable explanations can be obtained through the analysis results.

Because the TCMs for prevention and treatment of COVID-19 are mainly heat-clearing medicines, so the analysis focuses on the difference between the use of heat-clearing and non-heat-clearing medicines. Synthesizing the above statistical results, it turned out that in January, the utilization rate of heat-clearing and non-heat-clearing medicine in high and low-risk areas was significantly different (*p* < 0.01). In February, the north and south region were significantly different (*p* < 0.05), and in April, the high and low-risk area was significantly different (*p* < 0.01). According to the analysis, at the end of January, COVID-19 was just in the initial phase, and there was no effective prescription or decoction. Therefore, Chinese patent medicine became the main force to resist COVID-19 in high-risk areas. At the same time, the number of confirmed cases in low-risk areas has not yet risen to a severe level, so the Chinese patent medicines were not widely used. Consequently, different risk areas became the main factors affecting drug use in January. In mid-February, the number of confirmed cases nationwide peaked, and local treatment programmes began to be rolled out in each region, making it a major influence on drug use in February. In March, the epidemic situation was in a stage of significant decline, TCM has explored more mature and diversified treatment schemes, and the participation rate of Chinese patent medicines dropped. Hence, there was no significant difference among the three factors. In April, the epidemic situation was basically under control. At that time, the low-risk areas relaxed their vigilance, making the usage rate of heat-clearing medicine into the lowest point, so there was a significant difference with high-risk areas.

**FIGURE 3:**
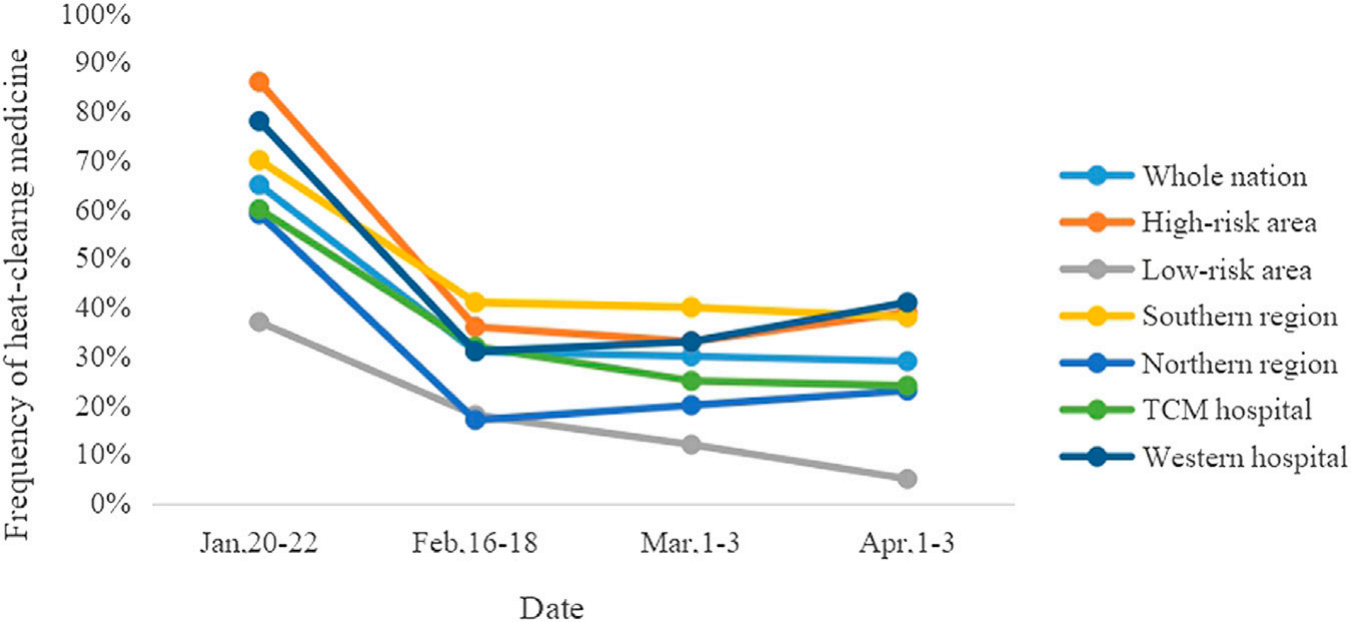
Changes in the utilization rate of heat-clearing medicine.

As shown in Figure 3, from January to April, the usage rate of heat-clearing medicines was the highest during January 20–22, when COVID-19 was just beginning to spread, while the remarkably decreased usage rates are shown in other three time points. Moreover, the usage rate of heat-clearing medicines in different risk areas, regions, and hospitals of different properties also showed the above trend, but did not show a significant correlation with the COVID-19 epidemic (*p* ＞ 0.05, Table 29). On account of the above results, the following considerations are made: 1) The Chinese patent medicines mainly participate in the early stage of the COVID-19, and the participation decreases in the outbreak stage, the decline stage, and the end-stage. 2) It is speculated that there were many blind purchases or use of Chinese patent medicines in the early stage, due to the public’s lack of understanding of COVID-19, panic mentality, and the pharmacy’s lax control over the use of heat-clearing medicines. 3) In this survey, the collected time points are limited with a short period, which cannot fully reflect the specific change rule of utilization rate over time. More evidence is needed to verify the above speculations further. 4) In correlation analysis, the sample size is too small to fully explain the relationship between the epidemic situation and three factors.

## Conclusion

At present, studies on Chinese patent medicines for COVID-19 mostly focus on therapeutic regimens, clinical observation, and pharmacological studies. There is almost no analysis of the overall use of Chinese patent medicines during COVID-19. This study was conducted to investigate the use of Chinese patent medicines in 24 third-grade class-A hospitals in 14 provinces or cities of China during the epidemic of COVID-19. And we have found that Chinese patent medicines play a role in the fight against COVID-19 and heat-clearing medicines were the most used weapons. Moreover, the risk area is the main influencing factor for the use of Chinese patent medicines.

Heat-clearing medicine, especially with the antiviral effect, has a high utilization rate during January-April, so it is considered that Chinese patent medicine has a certain degree of participation in the fight against COVID-19. On the whole, previous studies paid more attention to the medication difference of three-concerned therapy of “individual concerned therapy, environment concerned therapy, climate concerned therapy,” and this study confirmed that the use of Chinese patent medicine was different in the regions to some extent. Furthermore, two other factors were considered in the investigation, namely, hospitals of different properties and different risk regions. It was found that different risk regions were the main factor affecting the utilization rate of heat-clearing drugs.

On January 31, 2020, COVID-19 was listed as a public health emergency of international concern by the World Health Organization, which seriously endangered people’s health and public safety, and became one of the major epidemics after SARS in 2003 (Zhong et al., 2003; Cucinotta and Vanelli, 2020). So far, there is still no effective antiviral treatment for COVID-19 (Shereen et al., 2020; Stawicki et al., 2020). Symptomatic support therapy and comprehensive interventions are mainly used in clinical practice (Wu et al., 2020b).

Since the outbreak of COVID-19 (Wu et al., 2020b), TCM has been able to get involved in the whole process of treatment and achieved remarkable results (Li et al., 2020). TCM integrates its treatment principles (syndrome differentiation and three-concerned therapy of “individual concerned therapy, environment concerned therapy, climate concerned therapy”) (Wu et al., 2020a) with the different stages of COVID-19. Clinical studies (Liu et al., 2020; Zhang et al., 2020a) showed that TCM could improve the symptoms, shorten the course of treatment, and prevent conversion to the severe state for ordinary patients. For severe and critical patients, TCM can reduce pulmonary exudation, control inflammatory overreaction, improve oxygenation level, stabilize blood oxygen saturation, and reduce the use of hormones and antibiotics to prevent the deterioration of the disease. For convalescent patients, the rehabilitation process can be promoted by TCM. Meanwhile, patients with COVID-19 also include the elderly, children, pregnant women, and those with basic diseases, whose medications have also been considered in clinical treatment. Besides, the syndrome characteristics of COVID-19 in different regions are “the same but different.” Although the common characteristic is “wet,” different regions have diverse pathogenesis due to the environmental aspect (Ma et al., 2020; Shi et al., 2020) and other factors. Therefore, multiple TCM medication plans have been introduced in different regions in China. Moreover, many studies (Bashir et al., 2020; Chen et al., 2020; Tosepu et al., 2020) have shown that climate change can affect the spread of the COVID-19 and the pathogenesis of the human body, so the use of TCM should also take into account the influence of seasonal variations. Besides, some studies have suggested that the transmission of COVID-19 is related to air pollution and population density (Kadi and Khelfaoui, 2020; Martelletti and Martelletti, 2020). The cure rate and prognosis of COVID-19 are closely related to the underlying diseases such as cancer, hypertension, body mass index, and diabetes (Malik et al., 2020; Meng et al., 2020; Pugliese et al., 2020). These views have a high value of in-depth thinking and provide more direction for the research of drug use for COVID-19.

The TCMs involved in anti-epidemic include TCM decoction and Chinese patent medicine. It is well known that TCM decoction has played a great role in against the COVID-19 in China, while there are few reports and studies on Chinese patent medicine. The seventh Trial Version of the Guidelines for the Diagnosis and Treatment of COVID-19 by the National Health Commission of China recommends four oral Chinese patent medicines [Huoxiang Zhengqi capsule (pill, oral liquid, water), Jinhua Qinggan granule, Lianhua Qingwen capsule (granule), Shufeng Jiedu capsule (granule)] and eight TCM injections [(Xiyanping injection, Tanreqing injection, Xuebijing injection, Reduning injection, Xingnaojing injection, Shengmai injection, Shenfu injection, Shenmai injection)] respectively during the medical observation and clinical treatment period. According to some Chinese experts consensus and clinical experience, the intervention with Chinese patent medicine during medical observation can cut off the development of the disease in advance (Bao et al., 2020; Jin et al., 2020). Chinese patent medicine mainly plays two roles: on the one hand, it can provide symptomatic treatment; on the other hand, it can help strengthen immunity to resist the attack of the virus, so as to “prevent infection before illness” and “prevent transmission after illness” (Xiong et al., 2020). Clinical observation showed that (Duan et al., 2020; Yao et al., 2020), Jinhua Qinggan granules can significantly relieve the clinical symptoms of fever, cough, fatigue, and expectoration in mild COVID-19 patients. Lianhua Qingwen granules can significantly improve fever, cough, expectoration, and anhelation in COVID-19 patients, whose antifebrile time and the time of viral nucleic acid test turning negative were comparable to oseltamivir. Pharmacology experiments found that (Wang et al., 2020a) Chinese patent medicine showed a direct antiviral effect, could improve the inflammation caused by a virus infection, and have the function of the two-way adjusting the immune system. Furthermore, it can also impede or delay cytokine storm through the immunoregulation and anti-inflammatory action (Luo et al., 2020) and can suppress the occurrence or development of pulmonary fibrosis effectively. Huoxiang Zhengqi relieves symptoms through anti-inflammatory effects. Lianhua Qingwen defends the lung from COVID-19 by inhibiting pro-inflammatory cytokine production. Shufeng Jiedu plays roles in the COVID-19 through multiple targets and inflammatory signaling pathways. Xuebijing injection can reduce multiple organ damage by anti-inflammatory and improving immune function (Tong et al., 2020).

However, there are some limitations in this study. Firstly, the investigation only counted the names of the top three Chinese patent medicines used by hospitals, ignoring the specific application amount, which made it challenging to conduct more in-depth statistical analysis. Secondly, the distribution of the 24 hospitals investigated in this study is uneven across the country, which may affect the rigor of analysis results although the frequency was weighted in the analysis. Finally, the limited time points with a short time quantum cannot fully reflect the specific change rules of utilization rate over time, which need further in-depth discussion.

## Data Availability

The raw data supporting the conclusions of this article will be made available by the authors, without undue reservation, to any qualified researcher.
