# Erratum: A Retrospective Study on the Use of Chinese Patent Medicine in 24 Medical Institutions for COVID-19 in China

**DOI:** 10.3389/fphar.2021.654045

**Published:** 2021-02-25

**Authors:** Frontiers Production Office

**Affiliations:** Frontiers Media SA, Lausanne, Switzerland

**Keywords:** Chinese patent medicine, COVID-19, application regularity, correlative factor, retrospective analysis

Due to a production error, an incorrect **Funding** statement was used.

The correct Funding statement is “This study was funded by the National Key R&D Program of China (NO. 2019YFC1712000); Subject: Development of international standards for services in clinical pharmaceutical affairs of Chinese Patent Medicine and dispensing education (NO. 2019YFC1712002).”

The publisher apologizes for the mistake. The original version of this article has been updated.

