# Supplementary material for: A Retrospective Study on the Use of Chinese Patent Medicine in 24 Medical Institutions for COVID-19 in China
Source: Front Pharmacol. 2020 Nov 30;11:574562. doi: 10.3389/fphar.2020.574562 (PMC7990099; doi:10.3389/fphar.2020.574562)
Supplement: Supplementary file 1 [file datasheet1.pdf]

Appendix 1

Table1 Frequently-used Traditional Chinese Medicine in 24 hospitals

Name	Frequency	Percentage
Lianhua Qingwen granule (capsule)	10	31%
Lanqin oral liquid	7	21%
Jinhua Qinggan granule	5	15%
Bailing capsule(tablet)	4	12%
Huachansu capsule(tablet)	3	9%
Naoxintong capsule	2	6%
Antivirus oral liquid	2	6%

Table2 Utilization rate of Chinese patent medicine in high-risk area

Name	Frequency	Percentage
Lianhua Qingwen granule (capsule)	9	20%
Lanqin oral liquid	7	16%
Jinhua Qinggan granule	5	11%
Bailing capsule(tablet)	3	7%
Huachansu capsule	2	5%
Antivirus oral liquid	2	5%
Banlangen granule	1	2%
Qingfei pill	1	2%
Xuanfei Zhisou mixture	1	2%
Feilike mixture	1	2%
Yupingfeng granule	1	2%
Qingqiao Kangdu granule	1	2%
Jingyin mixture	1	2%
Compound Xianzhuli liquid	1	2%
Shiwei Longdanhua granule	1	2%
Chonglian oral liquid	1	2%
Compound shuanghua tablet	1	2%
Pudilan antiphlogistic oral liquid	1	2%
Huangkui capsule	1	2%
Shufeng Jiedu capsule	1	2%
Shengxuebao mixture	1	2%
Maxing Huatan mixture	1	2%
Compound Daqing granule	1	2%

Table3 Utilization rate of Chinese patent medicine in low-risk area

**Name**			
Modified Shuanghuanglian oral liquid	Shensong Yangxin capsule	Rougan Hepi pill	Relinqing granule
Bailing capsule	Xuefu Zhuyu capsule	Naoxintong capsule	Zukamu granule
Yinxing Mihuan oral liquid	Guanxin Danshen dropping pill	Qingfei Huatan mixture	Suhuang Zhike capsule
Lianhua Qingwen granule	Jinshuibao pill	Huachansu tablet	Danqi soft capsule
Compound Danshen dropping pill	Zhizhu Kuanzhong capsule	Yimucao capsule	

Table4 Utilization rate of Chinese patent medicine in southern region

**Name**	**Frequency**	**Percentage**
Lianhua Qingwen granule(capsule)	6	21%
Lanqin oral liquid	4	14%
Bailingcapsule(tablet)	3	10%
Huachansucapsule	2	7%
Shufeng Jiedu capsule	1	3.4%
Antivirus oral liquid	1	3.4%
Shengxuebao mixture	1	3.4%
Pudilan antiphlogistic oral liquid	1	3.4%
Xuanfei Zhisou mixture	1	3.4%
Feilike mixture	1	3.4%
Maxing Huatan mixture	1	3.4%
Qingqiao Kangdu granule	1	3.4%
Jingyin mixture	1	3.4%
Chonglian oral liquid	1	3.4%
Compound Daqing granule	1	3.4%
Yupingfeng granule	1	3.4%
Huaier granule	1	3.4%
Naoxintong capsule	1	3.4%

Table5 Utilization rate of Chinese patent medicine in northern region

**Name**	**Frequency**	**Percentage**
Jinhua Qinggan granule	5	13%
Lianhua Qingwen granule	4	10%
Lanqin oral liquid	3	8%
Antivirus oral liquid	1	2.7%

Banlangen granule	1	2.7%
Compound Xianzhuli liquid	1	2.7%
Shiwei Longdanhua granule	1	2.7%
Zukamu granule	1	2.7%
Qingfei pill	1	2.7%
Compound shuanghua tablet	1	2.7%
Honghua Qinggan thirteen pill	1	2.7%
Suhuang Zhike capsule	1	2.7%
Huangkui capsule	1	2.7%
Qingfei Huatan mixture	1	2.7%
Relinqing granule	1	2.7%
Modified Shuanghuanglian oral liquid	1	2.7%
Huachansu tablet	1	2.7%
Bailingcapsule	1	2.7%
Shensong Yangxin	1	2.7%
Zhizhu Kuanzhong capsule	1	2.7%
Rougan Hepi pill	1	2.7%
Jinshuibao pill	1	2.7%
Peiyuan Tongnao capsule	1	2.7%
Yinxing Mihuan oral liquid	1	2.7%
Xuefu Zhuyu capsule	1	2.7%
Naoxintong capsule	1	2.7%
Compound Danshen dropping pill	1	2.7%
Guanxin Danshen dropping pill	1	2.7%
Danqi soft capsule	1	2.7%

Table6 Utilization rate of Chinese patent medicine in TCM hospital

**Name**	**Frequency**	**Percentage**
Lianhua Qingwengranule(capsule)	8	15%
Bailingcapsule(tablet)	4	7%
Lanqin oral liquid	3	6%
Naoxintong capsule	2	4%
Jinhua Qinggan granule	2	4%
Huachansu capsule	2	4%
Banlangen granule	1	2%
Antivirus oral liquid	1	2%
Shufeng Jiedu capsule	1	2%
Compound Daqing granule	1	2%
Jingyin mixture	1	2%
Qingqiao Kangdu granule	1	2%
Pudilan antiphlogistic oral liquid	1	2%
Chonglian oral liquid	1	2%
Qingfei pill	1	2%

Zukamu granule	1	2%
Huachansu tablet	1	2%
Huangkui capsule	1	2%
Qingfei Huatan mixture	1	2%
Maxing Huatan mixture	1	2%
Relinqing granule	1	2%
Honghua Qinggan thirteen pill	1	2%
Modified Shuanghuanglian oral liquid	1	2%
Suhuang Zhike capsule	1	2%
Xuanfei Zhisou mixture	1	2%
Yupingfeng granule	1	2%
Shengxuebao mixture	1	2%
Shensong Yangxin	1	2%
Jinshuibao pill	1	2%
Rougan Hepi pill	1	2%
Zhizhu Kuanzhong capsule	1	2%
Xuefu Zhuyu capsule	1	2%
Compound Danshen dropping pill	1	2%
Guanxin Danshen dropping pill	1	2%
Danqi soft capsule	1	2%
Yinxing Mihuan oral liquid	1	2%

Table7 Utilization rate of Chinese patent medicine in western medical hospital

**Name**	**Frequency**	**Percentage**
Lanqin oral liquid	4	23%
Jinhua Qinggan granule	3	17%
Lianhua Qingwen granule	2	12%
Antivirus oral liquid	1	7%
Compound Xianzhuli liquid	1	7%
Compound shuanghua tablet	1	7%
Shiwei Longdanhua granule	1	7%
Feilike mixture	1	7%
Huaier granule	1	7%
Peiyuan Tongnao capsule	1	7%
Kangfuxin liquid	1	7%

Table8 Frequently-used Traditional Chinese Medicine in 24 hospitals

**Name**	**Frequency**	**Percentage**
Bailingcapsule(tablet)	7	9%
Compound Danshen dropping pill	6	8%
Lianhua Qingwen	5	7%

capsule(granule)		
Naoxintong capsule	5	7%
Jinshuibao pill	4	5%
Huangkui capsule	2	3%
Compound Xueshuantong capsule	2	3%
Xinyuan capsule	2	3%
Shensong Yangxin	2	3%
Linaoxin tablet	2	3%
Tiandan Tongluo capsule	2	3%
Ginkgo tablet (drop pill)	2	3%

Table9 Utilization rate of Chinese patent medicine in high-risk area

**Name**	**Frequency**	**Percentage**
Lianhua Qingwengranule(capsule)	4	7%
Bailingcapsule(tablet)	4	7%
Compound Danshen dropping pill	4	7%
Jinshuibao pill	3	5%
Naoxintong capsule	2	3%
Linaoxin tablet	2	3%
Xinyuan capsule	2	3%
Tiandan Tongluo capsule	2	3%
Xuebijing injection	1	2%
Xiyanping injection	1	2%
Jinhua Qinggan granule	1	2%
Lanqin oral liquid	1	2%
Chonglian oral liquid	1	2%
Jingyin mixture	1	2%
Banlangen granule	1	2%
Huangkui capsule	1	2%
Compound Daqing granule	1	2%
Maxing Huatan mixture	1	2%
Feilike mixture	1	2%
Compound Huangqi jiedu mixture	1	2%
Honghua Qinggan pill	1	2%
Babaodan capsule	1	2%
Yindan Xinnaotong soft capsule	1	2%
Tongluo Yiqi pill	1	2%
Naoshuantong capsule	1	2%
Xiaoshuan Changyong capsule	1	2%
Qili Qiangxin capsule	1	2%
Xiaoshuan Tongluo capsule	1	2%
Xueshuan Xinmaining tablet	1	2%
Sanqi Shutong capsule	1	2%
Xiongdan capsule	1	2%
Ginkgo drop pill	1	2%

Xueshuantong granule	1	2%
Compound Xueshuantong capsule	1	2%
Shexiang Baoxin pill	1	2%
Yupingfeng granule	1	2%
Peiyuan Tongnao capsule	1	2%
Shenyan Kangfu tablet	1	2%
Zhenyuan capsule	1	2%
Kangfuxin liquid	1	2%
Yixinshu capsule	1	2%
Shenqi Gankang capsule	1	2%

Table10 Utilization rate of Chinese patent medicine in low-risk area

**Name**	**Frequency**	**Percentage**
Bailing capsule	3	14%
Naoxintong capsule	3	14%
Compound Danshen dropping pill	2	9%
Shensong Yangxin capsule	2	9%
Lianhua Qingwen granule	1	4.5%
Qingfei Huatan mixture	1	4.5%
Huangkui capsule	1	4.5%
Qishen Yiqi drop pill	1	4.5%
Wenxin granule	1	4.5%
Jinshuibao pill	1	4.5%
Shenkangfu capsule 2	1	4.5%
Ginkgo tablet	1	4.5%
Tongxinluo capsule	1	4.5%
Xuefu Zhuyu granule	1	4.5%
Yuxuebi capsule	1	4.5%
Compound Xueshuantong capsule	1	4.5%

Table11 Utilization rate of Chinese patent medicine in southern region

**Name**	**Frequency**	**Percentage**
Bailingcapsule(tablet)	4	11%
Lianhua Qingwengranule(capsule)	3	8%
Compound Danshen dropping pill	3	8%
Naoxintong capsule	2	5%
Xiyanping injection	1	3%

Xuebijing injection	1	3%
Lanqin oral liquid	1	3%
Chonglian oral liquid	1	3%
Jingyin mixture	1	3%
Compound Huangqi jiedu mixture	1	3%
Compound Daqing granule	1	3%
Maxing Huatan mixture	1	3%
Feilike mixture	1	3%
Honghua Qinggan pill	1	3%
Babaodan capsule	1	3%
Ginkgo drop pill	1	3%
Shexiang Baoxin pill	1	3%
Yindan Xinnaotong soft capsule	1	3%
Naoshuantong capsule	1	3%
Tiandan Tongluo capsule	1	3%
Tongluo Yiqi pill	1	3%
Xiaoshuan Changyong capsule	1	3%
Xiongdan capsule	1	3%
Jinshuibao pill	1	3%
Yixinshu capsule	1	3%
Yupingfeng granule	1	3%
Shenqi Duotang oral liquid	1	3%
Shenyan Kangfu tablet	1	3%
Shenqi Gankang capsule	1	3%

Table12 Utilization rate of Chinese patent medicine in northern region

**Name**	**Frequency**	**Percentage**
Bailing capsule	3	7%
Naoxintong capsule	3	7%
Jinshuibao pill	3	7%
Compound Danshen dropping pill	3	7%
Lianhua Qingwengranule	2	5%
Huangkui capsule	2	5%
Xinyuan capsule	2	5%

Shensong Yangxin	2	5%
Linaoxin tablet	2	5%
Compound Xueshuantong capsule	2	5%
Jinhua Qinggan granule	1	2%
Qingfei Huatan mixture	1	2%
Banlangen granule	1	2%
Ginkgo tablet	1	2%
Xuefu Zhuyugranule	1	2%
Xueshuantong granule	1	2%
Tongxinluo capsule	1	2%
Qili Qiangxin capsule	1	2%
Sanqi Shutong capsule	1	2%
Yuxuebi capsule	1	2%
Tiandan Tongluo capsule	1	2%
Xiaoshuan Tongluo capsule	1	2%
Xueshuan Xinmaining tablet	1	2%
Kangfuxin liquid	1	2%
Zhenyuan capsule	1	2%
Wenxin granule	1	2%
Qishen Yiqi drop pill	1	2%
Shenkangfu capsule 2	1	2%
Peiyuan Tongnao capsule	1	2%

Table13 Utilization rate of Chinese patent medicine in TCM hospital

Name	Frequency	Percentage
Bailingcapsule(tablet)	7	12%
Compound Danshen dropping pill	5	8%
Lianhua Qingwengranule(capsule)	4	7%
Naoxintong capsule	4	7%
Jinshuibao pill	3	5%
Huangkui capsule	2	3%
Shensong Yangxin	2	3%
Compound Xueshuantong capsule	2	3%
Xiyanping injection	1	2%
Xuebijing injection	1	2%
Maxing Huatan mixture	1	2%
Jingyin mixture	1	2%
Chonglian oral liquid	1	2%

Qingfei Huatan mixture	1	2%
Compound Daqing granule	1	2%
Honghua Qinggan pill	1	2%
Compound Huangqi jiedu mixture	1	2%
Babaodan capsule	1	2%
Ginkgo tablet	1	2%
Tongxinluo capsule	1	2%
Tongluo Yiqi pill	1	2%
Linaoxin tablet	1	2%
Tiandan Tongluo capsule	1	2%
Naoshuantong capsule	1	2%
Xueshuantong granule	1	2%
Yuxuebi capsule	1	2%
Xiongdan capsule	1	2%
Shexiang Baoxin pill	1	2%
Xuefu Zhuyugranule	1	2%
Qili Qiangxin capsule	1	2%
Shenqi Duotang oral liquid	1	2%
Wenxin granule	1	2%
Yupingfeng granule	1	2%
Qishen Yiqi drop pill	1	2%
Peiyuan Tongnao capsule	1	2%
Shenyan Kangfu tablet	1	2%
Shenkangfu capsule 2	1	2%

Table14 Utilization rate of Chinese patent medicine in western medical hospital

**Name**	**Frequency**	**Percentage**
Xinyuan capsule	2	11%
Lianhua Qingwen granule	1	6%
Jinhua Qinggan granule	1	6%
Lanqin oral liquid	1	6%
Banlangen granule	1	6%
Feilike mixture	1	6%
Sanqi Shutong capsule	1	6%
Tiandan Tongluo capsule	1	6%
Xiaoshuan Tongluo capsule	1	6%
Xueshuan Xinmaining tablet	1	6%
Linaoxin tablet	1	6%
Yindan Xinnaotong soft capsule	1	6%

Jinshuibao pill	1	6%
Kangfuxin liquid	1	6%
Zhenyuan capsule	1	6%
Yixinshu capsule	1	6%

Table15 Frequently-used Traditional Chinese Medicine in 24 hospitals

Name	Frequency	Percentage
Bailingcapsule(tablet)	7	9%
Compound Danshen dropping pill	6	7%
Naoxintong capsule	5	6%
Jinshuibao pill	4	5%
Shensong Yangxin	4	5%
Lianhua Qingwen granule(capsule)	4	5%
Huangkui capsule	3	4%
Compound Xueshuantong capsule	2	2%
Ginkgo drop pill(tablet)	2	2%

Table16 Utilization rate of Chinese patent medicine in high-risk area

Name	Frequency	Percentage
Bailing capsule(tablet)	6	9%
Lianhua Qingwen granule(capsule)	4	6%
Compound Danshen dropping pill	4	6%
Jinshuibao pill	4	6%
Naoxintong capsule	4	6%
Huangkui capsule	3	5%
Shensong Yangxin	2	3%
Ginkgo tablet (drop pill)	2	3%
Xiyanping injection	1	1.6%
Xuebijing injection	1	1.6%
Jinhua Qinggan granule	1	1.6%
Lanqin oral liquid	1	1.6%
Compound Huangqi jiedu mixture	1	1.6%
Compound Daqing granule	1	1.6%
Antivirus oral liquid	1	1.6%
Huachansucapsule	1	1.6%
Maxing Huatan mixture	1	1.6%
Honghua Qinggan pill	1	1.6%

Feilike mixture	1	1.6%
Zhenbao pill	1	1.6%
Shengxuebao mixture	1	1.6%
Shenqi Duotang oral liquid	1	1.6%
Dengzhan Shengmaicapsule	1	1.6%
Xinyuan capsule	1	1.6%
Wenxin granule	1	1.6%
Linglingcapsule	1	1.6%
Xintong oral liquid	1	1.6%
Shenqi Gankang capsule	1	1.6%
Yishen Huashi granule	1	1.6%
Congrong Yishen granule	1	1.6%
Compound Congrong Yizhicapsule	1	1.6%
Shenyan Kangfu tablet	1	1.6%
Shenshuainingcapsule	1	1.6%
Xuefu Zhuyu capsule	1	1.6%
Compound Xueshuantong capsule	1	1.6%
Xueshuantong granule	1	1.6%
Qili Qiangxin capsule	1	1.6%
Sanqi Shutongcapsule	1	1.6%
Xueshuan Xinmaining tablet	1	1.6%
Xiaoshuan Changyong capsule	1	1.6%
Linaoxin tablet	1	1.6%
Tiandan Tongluo capsule	1	1.6%
Xiongdan capsule	1	1.6%

Table17 Utilization rate of Chinese patent medicine in low-risk area

Name	Frequency	Percentage
Bailing capsule	2	10%
Compound Danshen dropping pill	2	10%
Shensong Yangxin	2	10%
Yifei Jiedu granule	1	5%
Yichuanpingcapsule	1	5%
Naoxintong capsule	1	5%
Yuxuebi capsule	1	5%
Danqicapsule	1	5%
Tongxinluo capsule	1	5%
Xuefu Zhuyu capsule	1	5%

Compound Xueshuantong capsule	1	5%
Jinshuibao pill	1	5%
Shenkangfu capsule 2	1	5%
Xianling Gubaocapsule	1	5%
Fufang Xuanjucapsule	1	5%
Rougan Hepi pill	1	5%
Zhizhu Kuanzhong capsule	1	5%

Table18 Utilization rate of Chinese patent medicine in southern region

Name	Frequency	Percentage
Lianhua Qingwengranule(capsule)	3	8%
Bailingcapsule(tablet)	3	8%
Naoxintong capsule	3	8%
Compound Danshen dropping pill	3	8%
Xuebijing injection	1	2.6%
Xiyanping injection	1	2.6%
Lanqin oral liquid	1	2.6%
Antivirus oral liquid	1	2.6%
Compound Huangqi jiedu mixture	1	2.6%
Compound Daqing granule	1	2.6%
Maxing Huatan mixture	1	2.6%
Huachansu capsule	1	2.6%
Honghua Qinggan pill	1	2.6%
Huangkui capsule	1	2.6%
Feilike mixture	1	2.6%
Jinshuibao pill	1	2.6%
Shengxuebao mixture	1	2.6%
Dengzhan Shengmai capsule	1	2.6%
Lingling capsule	1	2.6%
Shenqi Duotang oral liquid	1	2.6%
Wenxin granule	1	2.6%
Congrong Yishen granule	1	2.6%
Yishen Huashi granule	1	2.6%
Shenqi Gankang capsule	1	2.6%

Ginkgo drop pill	1	2.6%
Xiaoshuan Changyong capsule	1	2.6%
Tiandan Tongluo capsule	1	2.6%
Shenshuainingcapsule	1	2.6%
Shenyan Kangfu tablet	1	2.6%
Xiongdan capsule	1	2.6%

Table19 Utilization rate of Chinese patent medicine in northern region

Name	Frequency	Percentage
Bailing capsule	4	9%
Shensong Yangxin	4	9%
Jinshuibao pill	3	7%
Compound Danshen dropping pill	3	7%
Naoxintong capsule	2	5%
Huangkui capsule	2	5%
Compound Xueshuantong capsule	2	5%
Lianhua Qingwen granule	1	2.3%
Jinhua Qinggan granule	1	2.3%
Yifei Jiedu granule	1	2.3%
Yichuanpingcapsule	1	2.3%
Zhenbao pill	1	2.3%
Ginkgo tablet	1	2.3%
Danqi capsule	1	2.3%
Sanqi Shutong capsule	1	2.3%
Xueshuantong granule	1	2.3%
Tongxinluo capsule	1	2.3%
Xueshuan Xinmaining tablet	1	2.3%
Qili Qiangxin capsule	1	2.3%
Linaoxin tablet	1	2.3%
Yuxuebi capsule	1	2.3%
Xintong oral liquid	1	2.3%
Xuefu Zhuyu capsule	1	2.3%
Xinyuan capsule	1	2.3%

Xianling Gubao capsule	1	2.3%
Shenkangfu capsule 2	1	2.3%
Fufang Xuanju capsule	1	2.3%
Compound Congrong Yizhi capsule	1	2.3%
Zhizhu Kuanzhong capsule	1	2.3%
Rougan Hepi pill	1	2.3%

Table20 Utilization rate of Chinese patent medicine in TCM hospital

**Name**	**Frequency**	**Percentage**
Bailingcapsule(tablet)	5	8%
Compound Danshen dropping pill	5	8%
Naoxintong capsule	4	7%
Lianhua Qingwengranule(capsule)	3	5%
Jinshuibao pill	3	5%
Shensong Yangxin capsule	3	5%
Compound Xueshuantong capsule	2	3%
Ginkgo drop pill (tablet)	2	3%
Xiyanping injection	1	2%
Xuebijing injection	1	2%
Compound Daqing granule	1	2%
Compound Huangqi jiedu mixture	1	2%
Maxing Huatan mixture	1	2%
Yichuanping capsule	1	2%
Yifei Jiedu granule	1	2%
Honghua Qinggan pill	1	2%
Huachansu capsule	1	2%
Danqi capsule	1	2%
Xuefu Zhuyu capsule	1	2%
Yuxuebi capsule	1	2%
Tongxinluo capsule	1	2%
Xiaoshuan Changyong capsule	1	2%
Sanqi Shutongcapsule	1	2%
Tiandan Tongluo capsule	1	2%
Xueshuantong granule	1	2%
Qili Qiangxin capsule	1	2%
Xiongdan capsule	1	2%
Shenqi Duotang oral liquid	1	2%
Lingling capsule	1	2%
Shengxuebao mixture	1	2%
Wenxin granule	1	2%

Shenqi Gankang capsule	1	2%
Zhizhu Kuanzhong capsule	1	2%
Rougan Hepi pill	1	2%
Shenshuaining capsule	1	2%
Shenyan Kangfu tablet	1	2%
Shenkangfu capsule 2	1	2%
Fufang Xuanju capsule	1	2%
Xianling Gubao capsule	1	2%
Yishen Huashi granule	1	2%

Table21 Utilization rate of Chinese patent medicine in western medical hospital

Name	Frequency	Percentage
Bailing capsule	2	11%
Lianhua Qingwen granule	1	5.2%
Jinhua Qinggan granule	1	5.2%
Lanqin oral liquid	1	5.2%
Antivirus oral liquid	1	5.2%
Zhenbao pill	1	5.2%
Feilike mixture	1	5.2%
Compound Danshen dropping pill	1	5.2%
Naoxintong capsule	1	5.2%
Linaoxin tablet	1	5.2%
Xueshuan Xinmaining tablet	1	5.2%
Xintong oral liquid	1	5.2%
Dengzhan Shengmai capsule	1	5.2%
Jinshuibao pill	1	5.2%
Shensong Yangxin capsule	1	5.2%
Congrong Yishen granule	1	5.2%
Compound Congrong Yizhi capsule	1	5.2%
Xinyuan capsule	1	5.2%

Table22 Frequently-used Traditional Chinese Medicine in 24 hospitals

Name	Frequency	Percentage
Bailing capsule	7	16%
Compound Danshen dropping pill	6	14%

Naoxintong capsule	5	11%
Jinshuibao pill	5	11%
Lianhua Qingwen granule(capsule)	4	9%
Huachansu capsule	2	5%
Tiandan Tongluo capsule	2	5%
Kangfuxin liquid	2	5%
Shensong Yangxin	2	5%
Lanqin oral liquid	2	5%
Maizhiling tablet	2	5%
Yuxuebi capsule	2	5%
Huangkui capsule	2	5%

Table23 Utilization rate of Chinese patent medicine in high-risk area

Name	Frequency	Percentage
Bailingcapsule(tablet)	6	10%
Compound Danshen dropping pill	4	6%
Naoxintong capsule	4	6%
Jinshuibao pill	4	6%
Lianhua Qingwen granule(capsule)	3	5%
Lanqin oral liquid	2	3%
Huachansu capsule	2	3%
Huangkui capsule	2	3%
Maizhiling tablet	2	3%
Tiandan Tongluo capsule	2	3%
Jinhua Qinggan granule	1	1.7%
Chonglian oral liquid	1	1.7%
Compound Daqing granule	1	1.7%
Jingyin mixture	1	1.7%
Feilike mixture	1	1.7%
Niuhuang Qingxin pill	1	1.7%
Longqing tablet	1	1.7%
Zhenbao pill	1	1.7%
Honghua Qinggan thirteen pill	1	1.7%
Shengxuebao mixture	1	1.7%
Shensong Yangxin	1	1.7%
Longlu capsule	1	1.7%
Mingmu Yanggan pill	1	1.7%
Jiuwei Zhenxin granule	1	1.7%
Weimaining capsule	1	1.7%
Lishukang capsule	1	1.7%
Qiwei Wenyang capsule	1	1.7%
Congrong Yishen granule	1	1.7%

Wenxin granule	1	1.7%
Shenyan Kangfu tablet	1	1.7%
Huoxue Tongmai capsule	1	1.7%
Salvia miltiorrhiza polyphenolic acid for injection	1	1.7%
Compound Xueshuantong capsule	1	1.7%
Xueshuan Xinmaining tablet	1	1.7%
Yindan Xinnaotong soft capsule	1	1.7%
Xiaoshuan Tongluo capsule	1	1.7%
Yuxuebi capsule	1	1.7%
Xiaoshuan Changyong capsule	1	1.7%
Qufeng Zhitong pill	1	1.7%
Honghua Xiaoyaotablet	1	1.7%

Table24 Utilization rate of Chinese patent medicine in low-risk area

**Name**	**Frequency**	**Percentage**
Compound Danshen dropping pill	2	10%
Lianhua Qingwen granule	1	5%
Zhizhu Kuanzhong capsule	1	5%
Bailingcapsule	1	5%
Shensong Yangxin	1	5%
Jinshuibao pill	1	5%
Fufang Xuanju capsule	1	5%
Gujin pill	1	5%
Kangfuxin liquid	1	5%
Xianling Gubao capsule	1	5%
Longlu pill	1	5%
Naoxintong capsule	1	5%
Tongxinluo capsule	1	5%
Guanxin Danshen dropping pill	1	5%
Yinxing Mihuan oral liquid	1	5%
Xuefu Zhuyu capsule	1	5%
Yuxuebi capsule	1	5%
Danqi soft capsule	1	5%
moxa stick	1	5%
Suxiao Jiuxin pill	1	

Table25 Utilization rate of Chinese patent medicine in southern region

**Name**	**Frequency**	**Percentage**
Bailing capsule(tablet)	5	13%
Compound Danshen dropping pill	4	11%

Naoxintong capsule	3	8%
Jinshuibao pill	2	5%
Lianhua Qingwen capsule	1	2.7%
Lanqin oral liquid	1	2.7%
Compound Daqing granule	1	2.7%
Chonglian oral liquid	1	2.7%
Jingyin mixture	1	2.7%
Feilike mixture	1	2.7%
Huachansu capsule	1	2.7%
Huangkui capsule	1	2.7%
Longqing tablet	1	2.7%
Weimaining capsule	1	2.7%
Shenyan Kangfu tablet	1	2.7%
Longlu capsule	1	2.7%
Lishukang capsule	1	2.7%
Qiwei Wenyang capsule	1	2.7%
Congrong Yishen granule	1	2.7%
Shengxuebao mixture	1	2.7%
Wenxin granule	1	2.7%
Honghua Xiaoyao tablet	1	2.7%
Qufeng Zhitong pill	1	2.7%
Tiandan Tongluo capsule	1	2.7%
Xiaoshuan Changyong capsule	1	2.7%
Yindan Xinnaotong soft capsule	1	2.7%

Table26 Utilization rate of Chinese patent medicine in northern region

Name	Frequency	Percentage
Lianhua Qingwen capsule	3	7%
Jinshuibao pill	3	7%
Naoxintong capsule	2	5%
Bailing capsule	2	5%
Compound Danshen dropping pill	2	5%
Kangfuxin liquid	2	5%
Shensong Yangxin	2	5%
Yuxuebi capsule	2	5%
Jinhua Qinggangranule	1	2%
Huangkui capsule	1	2%
Lanqin oral liquid	1	2%
Yinxing Mihuan oral liquid	1	2%
Huachansu capsule	1	2%
Zhenbao pill	1	2%
Honghua Qinggan thirteen pill	1	2%
Niuhuang Qingxin pill	1	2%

Xianling Gubao capsule	1	2%
Longlu pill	1	2%
Gujin pill	1	2%
Fufang Xuanju capsule	1	2%
Mingmu Yanggan pill	1	2%
Jiuwei Zhenxin granule	1	2%
Zhizhu Kuanzhong capsule	1	2%
Compound Xueshuantong capsule	1	2%
Salvia miltiorrhiza polyphenolic acid for injection	1	2%
Xuefu Zhuyu capsule	1	2%
Danqi soft capsule	1	2%
Xueshuan Xinmaining tablet	1	2%
Tiandan Tongluo capsule	1	2%
Xiaoshuan Tongluo capsule	1	2%
Huoxue Tongmaicapsule	1	2%
Tongxinluo capsule	1	2%
Suxiao Jiuxin pill	1	2%
Guanxin Danshen dropping pill	1	2%
moxa stick	1	2%

Table27 Utilization rate of Chinese patent medicine in TCM hospital

Name	Frequency	Percentage
Bailingcapsule(tablet)	7	12%
Jinshuibao pill	5	9%
Naoxintong capsule	4	7%
Compound Danshen dropping pill	4	7%
Lianhua Qingwen capsule(granule)	2	3%
Huangkui capsule	2	3%
Shensong Yangxin	2	3%
Yuxuebi capsule	2	3%
Longlu capsule(pill)	2	3%
Chonglian oral liquid	1	2%
Jingyin mixture	1	2%
Huachansu capsule	1	2%
Compound Daqing granule	1	2%
Honghua Qinggan thirteen pill	1	2%
Longqing tablet	1	2%
Weimaining capsule	1	2%
Zhizhu Kuanzhong capsule	1	2%
Shengxuebao mixture	1	2%
Wenxin granule	1	2%
Shenyan Kangfu tablet	1	2%

Xianling Gubao capsule	1	2%
Lishukang capsule	1	2%
Fufang Xuanju capsule	1	2%
Mingmu Yanggan pill	1	2%
Gujin pill	1	2%
Kangfuxin liquid	1	2%
Danqi soft capsule	1	2%
Honghua Xiaoyao tablet	1	2%
Salvia miltiorrhiza polyphenolic acid for injection	1	2%
Guanxin Danshen dropping pill	1	2%
Tongxinluo capsule	1	2%
Tiandan Tongluo capsule	1	2%
Xuefu Zhuyu capsule	1	2%
Compound Xueshuantong capsule	1	2%
Suxiao Jiuxin pill	1	2%
Yinxing Mihuan oral liquid	1	2%
moxa stick	1	2%

Table28 Utilization rate of Chinese patent medicine in western medical hospital

**Name**	**Frequency**	**Percentage**
Lianhua Qingwen granule	2	11%
Lanqin oral liquid	2	11%
Jinhua Qinggan granule	1	5.2%
Huachansu capsule	1	5.2%
Niuhuang Qingxin pill	1	5.2%
Zhenbao pill	1	5.2%
Feilike mixture	1	5.2%
Qiwei Wenyang capsule	1	5.2%
Congrong Yishen granule	1	5.2%
Kangfuxin liquid	1	5.2%
Jiuwei Zhenxin granule	1	5.2%
Compound Danshen dropping pill	1	5.2%
Huoxue Tongmai capsule	1	5.2%
Xueshuan Xinmaining tablet	1	5.2%
Xiaoshuan Tongluo capsule	1	5.2%
Tiandan Tongluo capsule	1	5.2%
Yindan Xinnaotong soft capsule	1	5.2%
